# Preparation and Properties of Microcrystalline Cellulose/Fish Gelatin Composite Film

**DOI:** 10.3390/ma13194370

**Published:** 2020-09-30

**Authors:** Ling Pan, Peng Li, Yubo Tao

**Affiliations:** 1 College of Material Science and Engineering, Northeast Forestry University, Harbin 150040, China; panling@nefu.edu.cn; 2 State Key Laboratory of Biobased Material and Green Papermaking, Qilu University of Technology, Shandong Academy of Sciences, Jinan 250353, China

**Keywords:** fish gelatin, microcrystalline cellulose, film, biopolymers, ultrasound

## Abstract

As a natural macromolecule-based biomaterial, fish gelatin is used in medical materials for its low pathogen infection risk. However, because of poor mechanical properties, its application has been limited. In this study, microcrystalline cellulose-reinforced fish gelatin (FG/MCC) composite films were prepared with a biological cross-linking agent (genipin) under ultrasonic treatment. SEM micrographs showed that the smooth microstructure of FG film became increasingly disordered with the addition of MCC. The infrared spectrum analysis (FTIR) demonstrated the existence of hydrogen bond interaction between MCC and FG. Compared with the pure FG film, the tensile strength (TS) and modulus of elasticity (MOE) of composite films with MCC were improved, and the elongation at break (EAB) and swelling ratios (SR) were decreased. Ultrasonic treatment could further improve TS, MOE, and SR. When the composite film was prepared with 15% MCC and treated with ultrasound, the TS and MOE increased by 115% and 227%, respectively, while the EAB decreased by 35% and the SR decreased by 4% in comparison with pure FG films. Thermo-gravimetric analysis (TGA) showed that the FG/MCC composite films were stable below 100 °C. The above results indicate that the FG/MCC films have optimistic application prospects in the biomedical field.

## 1. Introduction

Chronic wounds caused by injuries, burns, and non-healing ulcers are a major unsolved clinical problem [1]. In recent years, various substitutes of tissue skin have been studied to treat skin wounds, many of which have been demonstrated to have significant repair consequences. The materials widely used for regeneration and skin repair mainly include synthetic and natural polymers. Natural polymers have similar properties to macromolecules that are metabolized and recognized by cell in the biological environment [2]. Collagen, in particular, is mainly used in the medical industry because of its excellent biological properties such as low immunity, promoting platelet aggregation, and cell proliferation [3]. However, the application is limited by its poor antibacterial performance, poor mechanical property, and fast degradation rate [4]. Traditionally, collagen has been isolated from the skins and bones of terrestrial mammals, such as cows and pigs [5]. However, its application has been restricted because of bovine spongiform encephalopathy (BSE) [6] and religious reasons [7]. Aquatic species have the advantages of lack of disease risk, lack of dietary restrictions, and high collagen yield and the processed by-products have been considered as potential alternative sources of collagen [8]. Warm-water fish (*Larimichthys polyactis* and *tilapia*) have higher imidic acid content (proline and hydroxyproline) [9] and more similar properties to porcine gelatin than cold-water fish (such as lower energy storage modulus and lower gelling and melting temperature) [10]. Swim bladders of *Larimichthys polyactis* are highly valued for their medicinal properties and as special adhesives as well as for clarifying wine, making them an excellent potential source of collagen [11]. It has been shown that the swim bladder, as a vital low-fat source for natural protein, is often used in medicine because of its low risk of pathogenic infection.

Furthermore, the gelatin extracted from aquatic organisms has been used as a biodegradable film material, which has high transparency and excellent barrier properties to gases, organic vapors, and oils [12]. However, as the FG film contains numerous hydrophilic amino acids with negligible or no sulfur-containing amino acids, it has the disadvantages of the water vapor barrier and poor water resistance [13]. Because the FG has various functional groups distributed along the main and side chains (amino and carboxyl groups), it can be further blended with other functional polymer molecules (such as hydroxyl groups of MCC) to improve the limitations of FG biopolymers (such as the poor bacteriostatic property, poor mechanical property, and fast degradation rate, etc., [8].

MCC has the advantages of rich sources, high mechanical properties, non-toxic, high surface area, and biocompatibility, achieving high application potentials in a number of areas, particularly as a reinforcement agent in polymer composites [14]. MCC has many unique properties that allow customization or grafting of chemical species and has been used as effective micron reinforcement for biopolymer films [15,16]. Studies have shown that MCC addition greatly enhanced the thermal and mechanical properties of biomaterials. In addition, compared with other natural polysaccharide polymers, MCC still has some disadvantages, (e.g., poor wetting ability, poor moisture absorption, and incompatibility with most of the polymer matrix) [17,18], resulting in aggregation, reunion, and other defects in the microcrystalline cellulose-reinforced fish gelatin (FG/MCC) blend materials. Moreover, the composites only mixed with MCC and FG have the disadvantages of low mechanical properties, easy to be damaged and fast degradation, especially in wet states. At present, the existing studies are all about the chemical modification of MCC, such as etherification, esterification, and chemical cross-linking of the two substrates. Common covalent cross-linkers (e.g., formaldehyde, glutaraldehyde, and epoxides) are still physiologically toxic to a certain extent because of the presence of by-products and unreacted parts produced during their synthetic reactions [19]. In a comparison, genipin, a potential natural cross-linking agent from *Gardenia jasminoides* Ellis, showed lower cytotoxicity and anti-inflammatory properties. Therefore, it has been used effectively in various amino-containing polymer molecules [20]. From physical point of view, downscaling the aggregation of MCC would result in better film quality. The ultrasonic technology can be used to improve the dispersion of MCC in the polymer matrix [21]. The acoustic cavitation formed during ultrasonic treatment can break the intermolecular and intra-molecular bonds of that leading to the fragmentation of clusters and aggregates [22], thus realizing a better dispersion of MCC in the matrix.

In recent years, there have been a large number of studies on the preparation and properties of FG films, but limited studies on the preparation of FG films from the swim bladder, and no studies on the properties of FG/MCC composite films from the swim bladder of *Larimichthys polyactis* were found during the literature review. Therefore, based on the principle of complementary advantages, FG/MCC composite films were prepared in this study to explore the effects of MCC contents and ultrasonic treatment on the morphology, swelling properties, mechanical properties, and thermal stability of composite films, providing a potential candidate material for the tissue engineering.

## 2. Materials and Methods

### 2.1. Materials

The FG extracted from the swim bladder of *Larimichthys polyactis* was purchased from Gao shunhang Co., Ltd. (Guangzhou, China) as described by Li et al. [23]. The MCC (α-Cellulose (C_6_H_10_O_5_) n, EC 232-674-9, CAS 9004-34-6, Molecular Weight 162.06) with a particle size less than 25 microns was provided by Aladdin Industrial Co., Ltd. (Shanghai, China). The glycerol with the purity of 99.0% was procured from Tianjin fuyu Fine Chemical Col., Ltd. (Tianjin, China). Genipin (C_11_H_14_O_5_, 98% purity, CAS 6902-77-8, Molecular Weight 226.23), white powder or white crystal, was supplied by Lin chuan zhixin Biotechnology Co., Ltd. (Fuzhou, China). All chemicals were of analytical grades.

### 2.2. Preparation of Composite Film

The FG was dissolved in distilled water to form a homogeneous solution of 5%, and then 30% glycerol (based on fish gelatin, *w*/*w*) was added as a plasticizer. After swelling at room temperature for 40 min, it was heated and stirred continuously in a water bath at 45 °C for 30 min until completely dissolved. Different masses of MCC were added to a certain amount of distilled water until they were fully swollen, making mixed solution dispersed by the ultrasound at 25 kHz, 100 W, and 50 min. The genipin solution of 1% was prepared at 37 °C. The above FG solution was mixed with the MCC solution with continuous stirring for 1 h in a water bath at 45 °C to obtain the final FG concentration of 3% accounting for the total mass of the solvent (water) in the FG/MCC mixture. After complete mixing, the mixed solution was cooled down at 37 °C and 2 mL genipin solution was added to the above FG/MCC solution. After cross-linking for 4 h with continuous slight stirring, four film-forming solutions: 5%, 10%, 15%, and 20% (MCC content on a dry FG basis, *w*/*w*) were obtained. Each mixture was divided into two parts, one of them was further processed by the ultrasound (25 kHz, 100 W, and 50 min). The composite films without ultrasonic treatment were labeled as FG/MCC 0, FG/MCC 5, FG/MCC 10, FG/MCC 15, and FG/MCC 20, respectively. The ultrasound-treated composite films were labeled as son-FG/MCC 5, son-FG/MCC 10, son-FG/MCC 15, and son-FG/MCC 20, respectively. The FG/MCC 0 film was used as the control group.

### 2.3. Film Casting, Drying, and Conditioning

The FG/MCC mixture was casted into a Teflon mold, then dried naturally at ambient temperature. Before characterization, all samples were conditioned for one week in the desiccators containing the saturated NaCl solution (Relative Humidity, 75%). Five measurements for each sample and the average were reported as a mechanical data value.

### 2.4. Characterization

The thickness of samples, was measured by digital electronic micrometer (Model 6636-IS3). The average of each sample was determined by ten locations taken discretionarily (accuracy of 1 µm).

The cross section morphology of the film was observed using the scanning electron microscopy (SEM, Hitachi TM-3030, Tokyo, Japan) QUANTA200. The cryo-fractured cross-section of the film was coated by gold spraying, and then observed under the condition of 1 kV acceleration voltage and 2000× amplification.

For the Fourier-transform infrared (FT-IR) measurement, the film was made into powder, pressed into KBr pellets, and then analyzed by a FT-IR spectrometer (Frontier, PerkinElmer Co., Ltd, Waltham, MA, USA) Nicolette 6700 in the range of 4000–500 cm^−1^.

In order to study the effects of MCC content and ultrasonic treatment on swelling properties of composite films, the swelling ratio of film in distilled water for a certain time was measured. The film was taken out after immersing in distilled water for a certain period of time at ambient temperature, and the water on the surface of film was gently wiped off with filter paper. Until the weight of the sample was constant, it reached a swelling equilibrium. The swelling ratios (*SR*) and equilibrium swelling ratios (*S_eq_*) can be calculated by the following formula:

(1) $$SR=(W_{t}-W_{0})/W_{0}$$

(2) $$S_{eq}=(W_{\infty }-W_{0})/W_{0}$$

where, $W_{0}$ and $W_{\infty }$ are the initial dry weight and constant weight at the swelling equilibrium, respectively. The $W_{t}$ is the weight of the film soaking in distilled water for a certain time duration. The reported value was the average of the three samples.

The stress–strain properties, such as the TS (MPa), EAB (%), and MOE (MPa), were determined as described by Pei et al. [24], performing on the Universal Testing Machine (CMT5504, Shenzhen SANS Test Machine Co. Ltd., Shenzhen, China) with a cross head speed of 3 mm/min. The mean value of ten films was taken as the mechanical data value of the sample. *TS*, *EAB*, and *MOE* were calculated according to the following equation:

(3) TS=F/ab

where F (N), a (mm), and b (mm) are breaking load, length, and width of sample, respectively.

(4) $$EAB=[\left(L_{1}-L_{0})/L_{0}\right]\times 100\%$$

where, $L_{0}$ and $L_{1}$ are the initial length (mm) and the length (mm) when fracture occurs in the film.

(5) MOE=σ/ε

where σ is the stress (MPa) and ε is the strain of the film.

TGA was conducted in the range of 40–800 °C using a thermo-gravimetric analyzer (Model 1100SF, Mettler Toledo international trading (Shanghai, China) co., Ltd., Shanghai, China) with a heating rate of 10 °C/min under the nitrogen atmosphere at a flow rate of 20 mL/min.

## 3. Results and Discussion

### 3.1. Thickness

**Figure 1:**
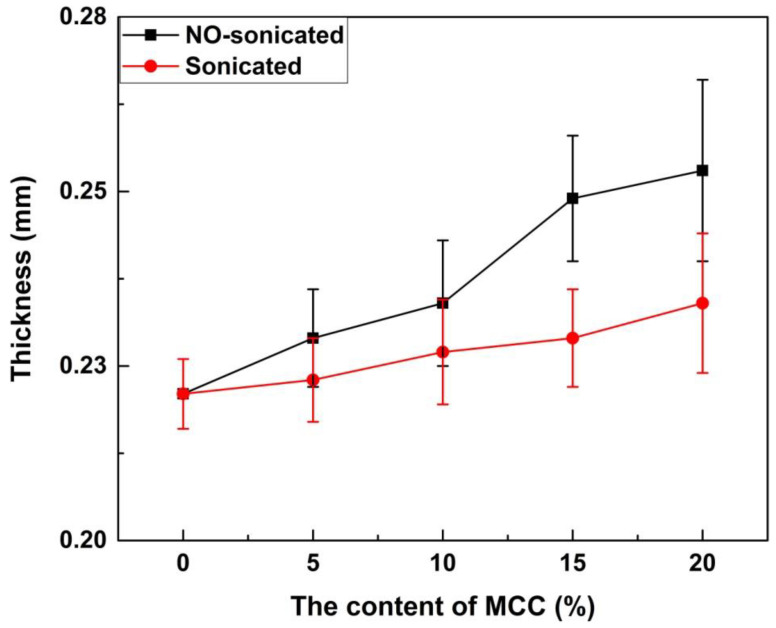
Thickness of microcrystalline cellulose-reinforced fish gelatin (FG/MCC) composite films with and without ultrasonic treatment.

The thicknesses of the film with different contents of MCC are shown in Figure 1. The thickness of the FG/MCC 0 film was the minimum (0.22 ± 0.005 mm), the FG/MCC 20 film was the maximum (0.25 ± 0.013 mm), and the other films were between 0.22 and 0.25 mm, showing that the thickness of film increased with the increase of the MCC content. The FG film had high density, because FG can arrange itself with less protrusion in the film matrix. The addition of MCC may cause agglomeration or irregular molecular alignment, resulting in the failure of the two different substances to form the compact and ordered film. In general, the thickness of film was determined by the composition of the film-forming solution and the properties of its components [25]. Arfat [26] and Ahmad [25,27] reported similar findings. The ultrasonic-treated film with the same amount of MCC was thinner than that of the un-treated film as shown in Figure 1. The ultrasonic treatment was conducive to the better dispersion of MCC in the FG matrix and promoted the interaction between FG and MCC in the composite film. Therefore, the composite film thickness decreased. Table 1 shows the density of the FG/MCC films with different MCC content. The density of all the composite films was about 1.3 g/cm^3^, and the addition of MCC had little effects on the density of the composite.

### 3.2. The Morphology of Film

**Figure 2:**
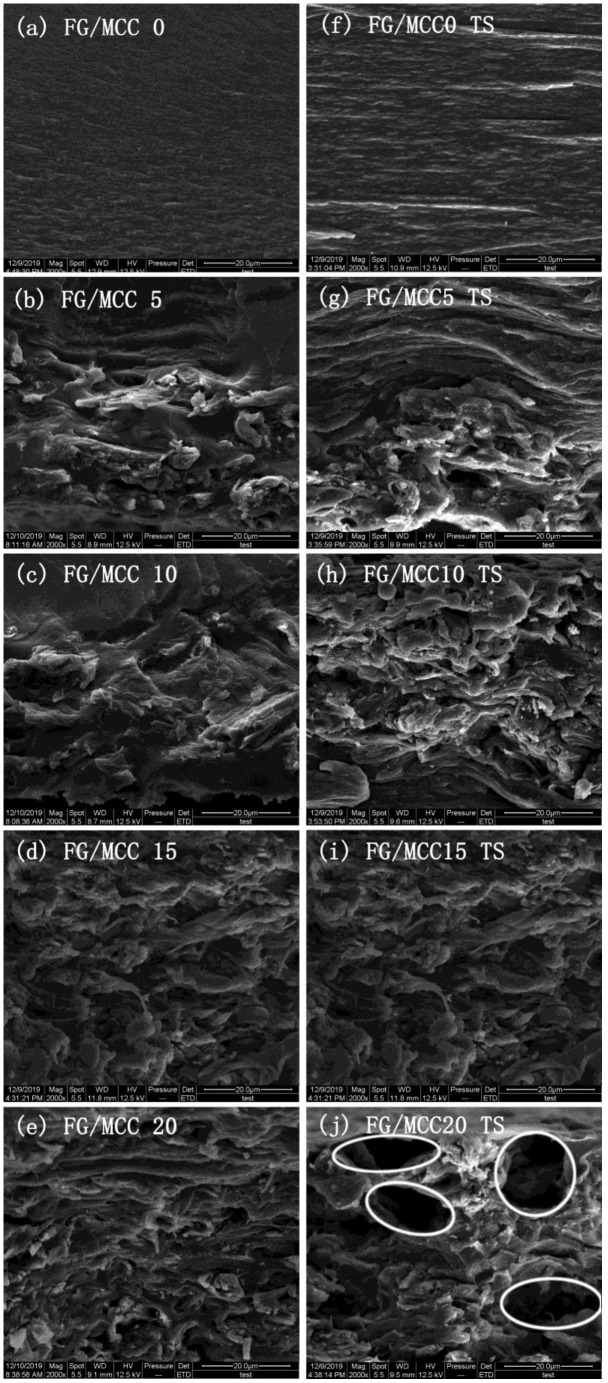
SEM images of the liquid nitrogen brittle section and tensile section (TS) of (**a**) FG/MCC, (**b**) 0 FG/MCC 5, (**c**) FG/MCC 10, (**d**) FG/MCC 15, (**e**) FG/MCC 20, (**f**) FG/MCC 0 TS, (**g**) FG/MCC 5 TS, (**h**) FG/MCC 10, (**i**) TS FG/MCC 15 and (**j**) FG/MCC 20.

**Figure 3:**
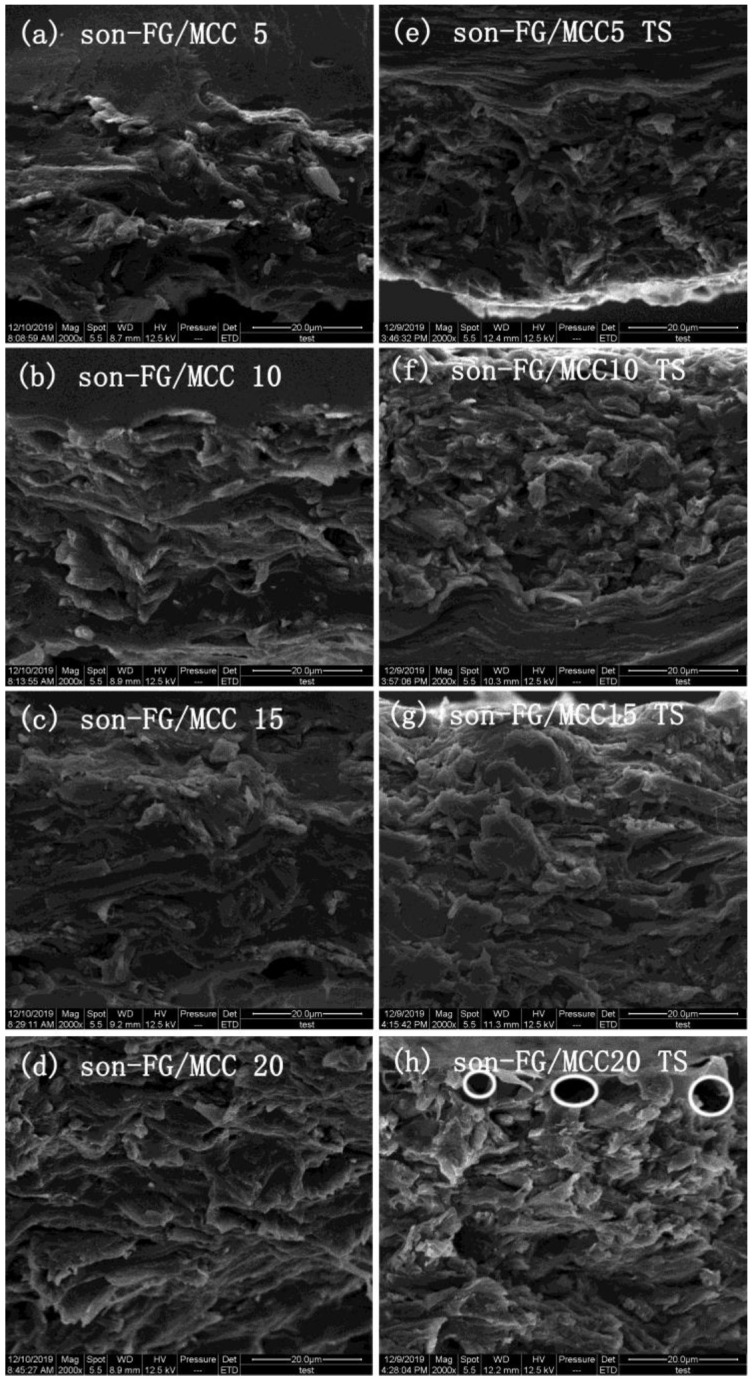
SEM images of the liquid nitrogen brittle section and tensile section (TS) of (**a**) son-FG/MCC 5, (**b**) son-FG/MCC 10, (**c**) son-FG/MCC 15, (**d**) son-FG/MCC 20, (**e**) son-FG/MCC 5 TS (**f**) son-FG/MCC 10 TS, (**g**) son-FG/MCC 15 TS and (**h**) son-FG/MCC 20 TS treated by ultrasound.

In order to further study the interaction between MCC and the FG matrix, the liquid nitrogen brittle section and tensile section of the film were observed by SEM. The section of the FG/MCC 0 film was relatively flat and uniform, while the cross section roughness of all the composite films with MCC added was increased. In the composite film, the FG/MCC 15 film had relatively fewer pores, and the roughness of the film cross section decreased slightly after the ultrasonic treatment under the same amount of MCC. The microstructure of composite films with different MCC contents were compared and analyzed in Figure 2 and Figure 3. As can be seen from the figures, the more MCC is added, the greater the roughness of the film. On the one side, excessive MCC was not conducive to dispersion in the FG matrix and the aggregation was generated. On the other side, it may also be related to the less interaction between FG and MCC molecules, resulting in a certain degree of microphase separation between MCC as dispersed phase and the FG as matrix. The cyro-fractured cross-sections of the composite films are shown in Figure 2b–e; the FG/MCC 15 (Figure 2d) film had fewer pores. The voids in the composite film increased when the MCC content increased to a higher concentration (20%), and the FG/MCC 20 (Figure 2e,j) film was the most obvious and its mechanical properties also decreased. The reason might be that, when two different types of substances were mixed together, the arrangement of FG and MCC molecules might occur in different ways during the film formation process, which resulted in excessive MCC overlapping with each other. In the wet film state, the void was filled by FG. After the natural drying or freeze-drying, the water in the film evaporated, resulting in a small number of holes and gaps.

After stretching, the FG/MCC 20 (Figure 2j) composite film was rougher with more holes and even cracks than that of others. This was because the dispersed phase of MCC and the FG matrix had different tolerances to external forces, and the phase separation was more obvious after the stretching. The TS of FG/MCC 20 (Figure 2j) was worse than that of the FG/MCC 15 (Figure 2i) film. The section morphology of the film was improved to some extent by the ultrasonic treatment, because ultrasound was more conducive to the dispersion of MCC in the FG matrix. Compared with the liquid nitrogen brittle section and tensile section of the FG/MCC 5 (Figure 2b,g), FG/MCC 10 (Figure 2c,h), FG/MCC 15 (Figure 2d,i), and FG/MCC 20 (Figure 2e,j) films, the surface roughness of son-FG/MCC 5 (Figure 3a,e), son-FG/MCC 10 (Figure 3b,f), son-FG/MCC 15 (Figure 3c,g), and son-FG/MCC 20 (Figure 3d,h) after the ultrasound treatment decreased. However, the low power of the ultrasonic irradiation resulted in the physical layer dispersion in MCC. There was no obvious difference between the SEM images before and after the ultrasonic treatment.

### 3.3. Attenuated Total Reflectance-Fourier Transforms Infrared Spectroscopy

**Figure 4:**
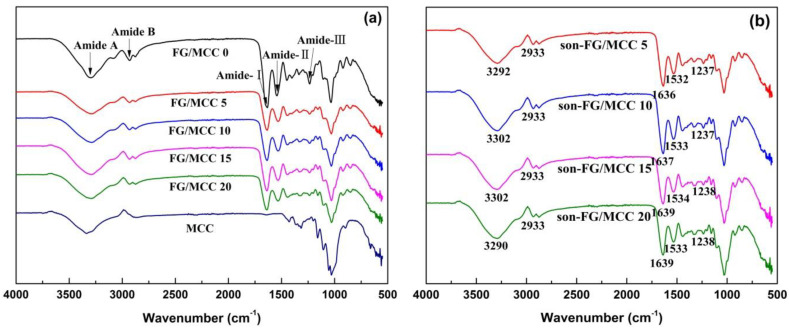
FTIR spectra of the FG/MCC films (**a**) and the son-FG/MCC films (**b**) treated by ultrasonic.

Figure 4 and Table 2 show the FTIR spectra of FG/MCC films and its characteristic peak. The characteristic peak of hydroxyl group in MCC was a wide peak, which was caused by the interaction of hydroxyl groups between different crystal types in MCC. It could also be explained that hydroxyl groups within or between molecules formed hydrogen bonds, which would lead to the shift/deviation of the characteristic peak of hydroxyl groups. Therefore, the characteristic peaks of hydroxyl groups at different offsets were superimposed to form a wide peak. As can be seen in Figure 4a, the stretching vibration absorption peak of the -OH group in MCC was around 3336 cm^−1^, and the absorption peak in FG/MCC films was 3289 cm^−1^ (Table 2), which moved in the direction of the lower wave number. This results indicated that -NH_2_ and -COOH groups in FG might form new hydrogen bonds with -OH group in MCC, resulting in deviation of the characteristic peak of the -OH group [28]. At the same time, the characteristic peaks of the -OH group became wider, which might be caused by the superposition of the -NH_2_ and -COOH- groups characteristic peaks in the protein and the –OH group characteristic peaks in MCC.

Peaks at 1634 cm^−1^, 1543 cm^−1^, and 1236 cm^−1^ respectively were the C=O band (amide-I) of the contraction vibration at 1680–1630 cm^−1^, the amide-II with N-H shock absorption and C-N stretching vibration at 1570–1510 cm^−1^, and the amide-III with C-N and the N-H stretching vibration absorption peak at 1335–1200 cm^−1^ [29]. FG/MCC composite films appeared at the FG characteristic peak, and some peaks moved slightly to lower or higher wave numbers with the addition of MCC. For instance, the absorption peak of amide-I (amide carbonyl in protein) and amide-III bands in the FG/MCC film slightly shifted to higher wave numbers. Since there was no carbonyl group in MCC, the deviation of the characteristic peak of carbonyl group in FG eliminated the factor of the peak superposition. The reason might be the introduction of cellulose component in FG and the formation of the intermolecular hydrogen bond. In the infrared spectrum, the vibration absorption peak intensity of the amide-II band in the composite film moved to lower wave number, and wave numbers were all 1532 cm^−1^, which was because the FG was introduced to the cellulose component, also formed a new hydrogen bonding interaction.

Figure 4b shows the ATR-FTIR spectra of FG/MCC films in the range of 4000–500 cm^−1^. Compared to the FG/MCC films without ultrasound treatment, the characteristic absorption peaks were not significantly changed, which might be due to the low power of ultrasonic cleaner and more physical dispersion effect on MCC, indicating that MCC still had the basic chemical structure of cellulose. In addition, continuous waves resulted in a slight change in the characteristic absorption peak [30], and the stretching vibration absorption peak of the −OH group moved from 3289 cm^−1^ to the high wave number. Compared to the un-ultrasonic-treated FG/MCC film, amide I, II, and III bands in the composite film with the ultrasonic treatment also slightly shifted to higher wave numbers, and the reason might be the conformational change caused by the ultrasonic treatment and the amide-cellulose interaction.

### 3.4. The Swelling Ratios and Equilibrium Swelling Ratios

**Figure 5:**
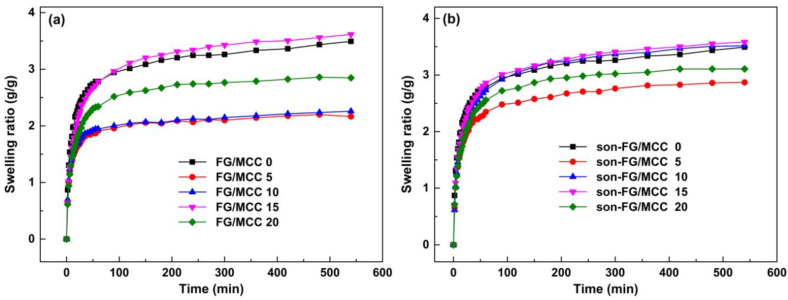
The swelling ratios of the films (**a**) and the ultrasonic films (**b**) immersed in distilled water with different time.

The nutrient needed for cell growth comes mainly from the tissue fluid around the wound at the early period of the wound healing, therefore, the water absorption of materials as a skin substitute is one of the important factors of biological activity in the clinic [31]. In addition, the water absorption is also a key factor for wound dressings to maintain a moist environment [32]. It can be seen from Figure 5 that all the films could rapidly absorb water and swell at 60 min, and basically close to their own swelling equilibrium, indicating that the swelling behavior of all films conformed to the diffusion mechanism [33]. The swelling property of the FG/MCC composite films were influenced to some extent by the addition of MCC. The reason was that carboxyl (–COOH) and amino groups (–NH_2_) in FG might form intermolecular hydrogen bonds with the hydroxyl groups (–OH) in MCC, which could effectively form network structure in the FG/MCC composite system, and limited the motion ability of the FG molecular chains, and reduced the SR of the composite films [27]. For composite films with MCC, the swelling property increased first and then decreased gradually in the range of 5 wt% to 20 wt% MCC. The reason why excessive MCC led to the decrease in SR might be the disturbance of the film-formation process in the presence of excessive MCC [31]. In Table 3, for un-ultrasonic-treated films, the S_eq_ of FG/MCC 15 (3.578) was the highest in the composite films with MCC added, but slightly less than FG/MCC 0 (3.767) film. The S_eq_ of the composite films with the same MCC content were increased after the ultrasonic treatment. Among them, the S_eq_ of son-FG/MCC 10 (3.614) and son-FG/MCC 15 (3.929) was close to and higher than that of the FG/MCC 0 film, respectively. The results confirmed that the ultrasonic treatment had a positive effect on swelling performance of composite films. In a word, the SR is largely determined by the water absorption and mechanism characteristics of the materials [34]. As a highly hydrophilic substance, the SR of FG was mainly dependent on the existence of hydrophilic groups and the cross-linking density of the composite materials due to its physical and chemical structures [35]. Therefore, FG/MCC 15, son-FG/MCC 10, and son-FG/MCC 15 were able to absorb sufficient nutrients to sustain the wound healing and early cell growth [36].

### 3.5. Mechanical Properties

Materials with good mechanical strength can promote the growth and proliferation of cells and facilitate tissue regeneration [37]. The mechanical properties of natural polymer materials, such as FG, are generally adequate. However, the enzymatic hydrolysis [38] and hydrolysis [39] of FG in vivo are too fast, which limit its wide clinical application.

**Figure 6:**
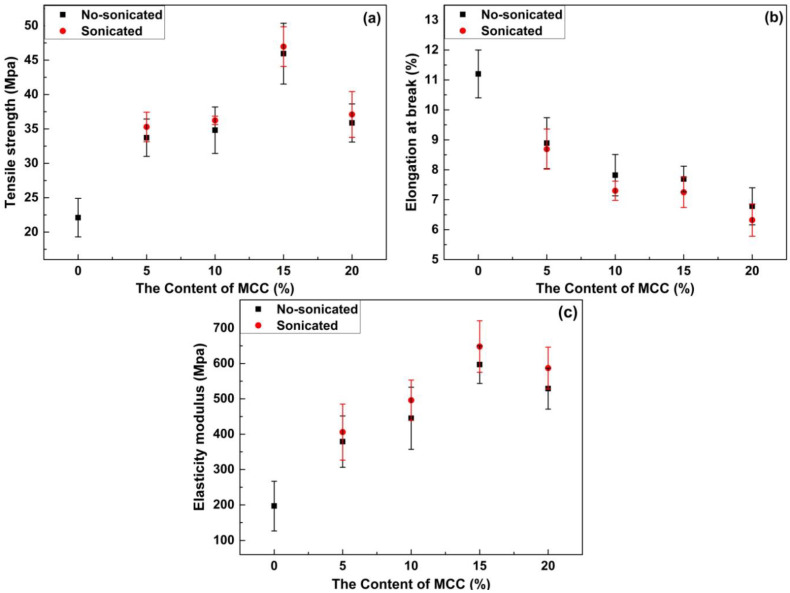
Tensile strength (**a**), elongation at break (**b**), and elasticity modulus tensile strength (**c**) for pure fish glue film and composite films reinforced with 0 wt% MCC, 5 wt% MCC, 10 wt% MCC, 15 wt% MCC, 20 wt% MCC and ultrasonic of that.

The mechanical properties of each material are shown in Figure 6 and Table 4. In general, the mechanical properties of the materials are closely related to the properties and chemical structures of the film-forming materials. Compared with the FG/MCC 0 film, the mechanical properties of the composite film can be greatly improved as shown in Figure 6. The reason was that the intermolecular hydrogen bond between the FG and MCC strengthened the interaction of the two substances. When an ideal wound dressing was used, high mechanical strength was the best choice [40]. In Figure 6a,c, with the addition of MCC, TS, and EAB of the FG/MCC composite film first increased and then decreased. With 5% MCC added, the mechanical properties of the composite films were improved clearly in comparison with FG film. However, when the MCC content reached 10%, the growth of the mechanical properties was not obvious. When the addition of MCC was 15%, the mechanical performance was the best. Compared with the FG/MCC 0 film (22.1 MPa), the TS (45.94 MPa) of the FG/MCC 15 composite film increased by about 2.1 times. The FG/MCC composite film can sustain much higher stress and was more rigid than the FG/MCC 0 film. The MCC molecule contained abundant hydroxyl groups, and the terminal -COOH group and -NH_2_ group in FG may form intermolecular hydrogen bonds with the -OH group in MCC [40,41]. This indicated that it had stronger mechanical properties than a single component. In addition, the mechanical property of the composite film depended not only on the enhanced property of micron fillers, but also on the interaction between micron fillers and matrix, as well as the dispersion in the matrix [42]. However, when the amount of MCC was 20%, the TS (35.87 MPa) and MOE (529 MPa) of the composite film began to decrease. The reason was that excessive MCC agglomerated when compounded with FG, thus affecting the structure order, so its mechanical property would be reduced. As observed on SEM images, it could be seen that the cross section of FG/MCC 20 became rougher and the holes were larger and more, which was also the reason for the reduction of its mechanical properties. With the increase of MCC, the EAB decreased. In this sense, more MCC content would reduce the cohesion of the film matrix and the resistance to rupture the film. The FG film had the highest EAB. This was attributed to the reason that the protein molecules combined with more water molecules acted as plasticizers for the film, resulting in a higher EAB. However, it was decreased with the increase in MCC as shown in Figure 6. When the amount of MCC was 20%, the EAB of the FG/MCC film was the lowest (6.78%). Therefore, high concentration of MCC destroyed the EAB of the composite film. In the study on the cellulose whisker-reinforced FG film by Talita et al. [30], the similar trend in mechanical properties was obtained, where the TS of the film was significantly improved when 5% cellulose whisker was added, but the performance began to decline when excessive cellulose whisker was added.

For ultrasonic treated films, the TS, EAB, and MOE had similar behaviors and trends as that of the untreated films. Compared with the film without the ultrasonic treatment, both TS and MOE of the ultrasonic treated films were improved accordingly under the same conditions. The reason can be attributed in part to the ultrasonic treatment that facilitated the even dispersion [43] and increased the surface area of the polymer [44]. In addition, the ultrasonic treatment may have an effect on the FG itself. According to the research of Liu, Teleez-Garay, and Castell-Perez [45], the conformational change on the peanut protein could increase the cross-linking of the disulfide bond and hydrophobic effect, which was beneficial to the tensile properties of the peanut protein film. The ultrasonic treatment might induce conformational change of the FG, leading to the improvement of mechanical properties. Compared with the film without the ultrasonic treatment, the EAB of the composite film was slightly reduced after the ultrasonic treatment.

### 3.6. Thermo-Gravimetric Analysis

**Figure 7:**
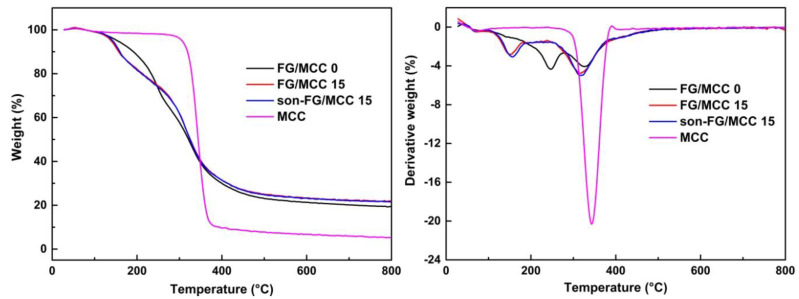
TGA and DTG curves of the FG/MCC 0, FG/MCC 15, and son-FG/MCC 15 composite films.

The TGA curves (Figure 7) showed the thermal degradation properties of the three films, aiming at investigating the influence of MCC on the thermal stability of the composite membrane and the interaction between the dispersed phase and the continuous phase. As shown in Figure 7, the addition of MCC had a slight effect on the initial degradation temperature (Ti) of the composite films. Compared with the FG film, when the weight loss of FG/MCC was 5% (T5), the degradation temperature dropped, and the range of decline was around 10–15 °C, and the maximum degradation temperature (Tf) also decreased. However, the Tf of the FG/MCC film and son-FG/MCC composite film was not significantly different, which were 137.6 °C and 142.9 °C, respectively. Compared with the FG film, the final residual carbon contents (R) of FG/MCC and son-FG/MCC composite films were increased, and the residual masses were 19.4%, 21.9%, and 21.5%, respectively, indicating that the cross-linking density between the dispersed phase (MCC) and the continuous phase was improved [41]. Compared with FG, the minor difference of carbon residue in composite film was mainly due to the composition of formula and type, blending ratio, non-covalent, interaction, and other factors. Therefore, TGA and DTG examinations indicated that the addition of MCC had a certain influence on the thermal stability of the FG/MCC composite film.

## 4. Conclusions

In this study, the FG/MCC composite film was prepared using a simple, green, and fast method, in which MCC was used as the reinforcing phase. The interfacial interaction between FG and MCC changed the properties of the collagen matrix. The composite film section with MCC was rough, and when more MCC was added, the film section obtained was more uneven. The ultrasonic treatment improved the surface roughness of the film to a certain extent. FTIR results showed that FG and MCC had strong hydrogen bond, and their molecular structure was constructed by non-covalent bond. FG/MCC composite films could expand rapidly by absorbing water in the short time and made them close to their own swelling equilibrium. The addition of MCC reduced the expansion rate of composite films, and the ultrasonic treatment increased the expansion rate of the composite films. However, the addition of MCC affected the thermal stability of FG/MCC composite films. Compared with the FG/ MCC 0 film, a certain amount of MCC could improve the TS and MOE of the composite film, but reduced the EAB of the film. The TS and MOE of the ultrasonic-treated samples (with the same MCC dosage) were also improved. In addition, these results indicated that FG/MCC composite films had great potential in biomedical applications.

## Figures and Tables

**Table 1:** The density (g/cm^3^) of the FG/MCC films with different MCC content.

Samples	FG/MCC 0	FG/MCC 5	FG/MCC 10	FG/MCC 15	FG/MCC 20
FG/MCC	1.3	1.3	1.3	1.4	1.3
Son-FG/MCC	/	1.3	1.4	1.4	1.3

**Table 2:** The Fourier-transform infrared spectra peaks of the FG/MCC composite films.

Samples	Amide-A/cm^−^^1^	Amide-B/cm^−^^1^	Amide-I/cm^−^^1^	Amide-Ⅱ/cm^−^^1^	Amide-III/cm^−^^1^
FG/MCC 0	3293	2933	1634	1543	1236
FG/MCC 5	3292	2933	1636	1532	1236
FG/MCC 10	3302	2933	1637	1533	1237
FG/MCC 15	3302	2933	1639	1533	1237
FG/MCC 20	3290	2933	1639	1533	1238
son-FG/MCC 5	3292	2933	1636	1532	1237
son-FG/MCC 10	3302	2933	1637	1533	1237
son-FG/MCC 15	3302	2933	1639	1534	1238
son-FG/MCC 20	3290	2933	1639	1533	1238
MCC	3336	-	-	-	-

**Table 3:** The equilibrium swelling ratios (S_eq_, *g/g*) of the FG/MCC films with different MCC content.

Samples	FG/MCC 0	FG/MCC 5	FG/MCC 10	FG/MCC 15	FG/MCC 20
FG/MCC	3.767	2.435	2.543	3.578	2.978
son-FG/MCC	/	3.415	3.614	3.929	3.131

**Table 4:** The tensile strength, elongation at break, and elasticity modulus tensile strength of pure fish glue film and composite films.

Samples	FG/MCC 0	FG/MCC 5	FG/MCC 10	FG/MCC 15	FG/MCC 20	Son-FG/MCC 5	Son-FG/MCC 10	Son-FG/MCC 15	Son-FG/MCC 20
TS (MPa)	22.1	33.72	34.81	45.94	35.87	35.29	36.24	46.96	37.1
EAB (%)	11.2	8.89	7.82	7.69	6.78	8.69	7.3	7.25	6.32
MOE (MPa)	197	379	445	597	529	406	496	648	587
